# Increased number of T cells and exacerbated inflammatory pathophysiology in a human IgG4 knock-in MRL/lpr mouse model

**DOI:** 10.1371/journal.pone.0279389

**Published:** 2023-02-10

**Authors:** Yoshie Gon, Tsugumitsu Kandou, Tatsuaki Tsuruyama, Takeshi Iwasaki, Koji Kitagori, Kosaku Murakami, Ran Nakashima, Shuji Akizuki, Akio Morinobu, Masaki Hikida, Tsuneyo Mimori, Hajime Yoshifuji

**Affiliations:** 1 Department of Rheumatology and Clinical Immunology, Graduate School of Medicine, Kyoto University, Kyoto, Japan; 2 Department of Clinical Immunology, Osaka Metropolitan University Graduate School of Medicine, Osaka, Japan; 3 Department of Drug Discovery Medicine, Pathology Division, Graduate School of Medicine, Kyoto University, Kyoto, Japan; 4 Center for Cancer Immunotherapy and Immunobiology, Graduate School of Medicine, Kyoto University, Kyoto, Japan; 5 Faculty of Engineering Science, Graduate School of Engineering Science, Akita University, Akita, Japan; 6 Ijinkai Takeda General Hospital, Kyoto, Japan; Universite Paris-Saclay, FRANCE

## Abstract

Immunoglobulin (Ig) G4 is an IgG subclass that can exhibit inhibitory functions under certain conditions because of its capacity to carry out Fab-arm exchange, inability to form immune complexes, and lack of antibody-dependent and complement-dependent cytotoxicity. Although several diseases have been associated with IgG4, its role in the disease pathogeneses remains unclear. Since mice do not express an IgG subclass that is identical to the human IgG4 (hIgG4), we generated h*IGHG4* knock-in (KI) mice and analyzed their phenotypes. To preserve the rearrangement of the variable, diversity, and joining regions in the IGH gene, we transfected a constant region of the h*IGHG4* gene into C57BL/6NCrSlc mice by using a gene targeting method. Although the mRNA expression of h*IGHG4* was detected in the murine spleen, the serum level of the hIgG4 protein was low in C57BL/6-IgG4KI mice. To enhance the production of IgG4, we established an MRL/lpr-IgG4KI mice model by backcrossing. These mice showed a high IgG4 concentration in the sera and increased populations of IgG4-positive plasma cells and CD3^+^B220^+^CD138^+^ T cells in the spleen. Moreover, these mice showed aggravated inflammation in organs, such as the salivary glands and stomach. The MRL/lpr-IgG4KI mouse model established in the present study might be useful for studying IgG4-related disease, IgG4-type antibody-related diseases, and allergic diseases.

## Introduction

Immunoglobulin (Ig) G plays a central role in the neutralization and opsonization of foreign antigens. There are four subclasses of IgG: IgG1, IgG2, IgG3, and IgG4. IgG1 and IgG2 can neutralize viral and bacterial exotoxins and bacterial polysaccharides [1]. IgG1 and IgG3 have high affinity for Fcγ receptors (FcγR) and assist dendritic cells in capturing antigens and promoting antigen presentation.

IgG4 antibodies are the least abundant of all IgG subclasses (about 5% of the IgG subclasses) in humans [2] and have unique characteristics that distinguish them from other IgG subclasses. First, IgG4 has low binding affinity for FcγR and complements, and thus, exhibits a lower ability to induce inflammation than the other subclasses [3, 4]. Furthermore, IgG4 has the ability to undergo Fab-arm exchange and can exchange a pair of heavy and light chains with another IgG4 molecule, thereby gaining two different antigen-binding sites [4]. Moreover, serum IgG4 levels are parallel with levels of IgE in allergic conditions. It is hence speculated that IgG4 neutralizes the binding between IgE and its antigen and inhibits the activation of mast cells and basophils [5]. IgG4 is an important marker of IgG4-related disease (IgG4-RD) and is a predominant subclass of autoantibodies found in pemphigus [6], myasthenia gravis [7], thrombotic thrombocytopenia [8], and idiopathic membranous nephropathy [9]. Furthermore, IgG4 is elevated in patients undergoing allergen-specific immunotolerance therapy [10]. Additionally, the IgE/IgG4 ratio is relevant to the diagnosis of food allergies [11].

In mice, IgG1 serves as the counterpart of human IgG4 (hIgG4). Mouse IgG1 (mIgG1) and hIgG4 induced by T-helper-2 cells bind weakly to C1q [12], are homologous in the hinge region, and have similar flexibility in antigen binding. In fact, studies have employed mIgG1 as a counterpart of hIgG4 in mouse models of pemphigoid [13], myasthenia gravis [14], and IgG4-RD [15]. However, mIgG1 and hIgG4 bind differently to FcγRIIb [16]; mIgG1 binds strongly to FcγRIIb, while hIgG4 binds weakly. In humans, IgG3 is the IgG subclass that binds strongly to FcγRIIb.

In this study, we aimed to investigate the function of hIgG4, whose function does not completely match that of mIgG1. Although there have been several studies that have attempted to express hIgG4 proteins into mice, there have been no reports of mice that express h*IGHG4*. Here, we established a hIgG4-knock-in (KI) mice model and analyzed the phenotypes. We observed that serum IgG4 level was increased and IgG4-positive plasma cells were identified in the spleen of MRL-lpr/hIgG4KI mice. The extent of inflammatory cell infiltration was greater and the percentage of CD3^+^B220^+^CD138^+^ cells was higher in MRL/lpr/hIgG4KI mice compared with those in control MRL/lpr mice. Our findings suggest that this newly established mouse model may be useful to study IgG4-RD, IgG4-type antibody-related diseases, and allergic diseases.

## Materials and methods

### Animals

C57BL/6NCrSlc and MRL/MpJJmsSlc-lpr/lpr mice were purchased from Japan SLC, Inc. (Shizuoka, Japan) and bred at Kyoto University hospital under specific pathogen-free conditions. The MRL/lpr mouse strain used in the present study (MRL/MpJJmsSlc-lpr/lpr) has an elevated lifespan compared to the MRL/MpJ-Fas^lpr^/J mouse strain. The survival rate of MRL/MpJJmsSlc-lpr/lpr mice at Japan SLC, Inc. is shown in S1 Fig in S1 File. We outsourced the generation of h*IGHG4* knock-in C57BL/6NCrSlc mice to Unitech Co. Ltd. (Chiba, Japan). We were granted approval (No. 130154) by the Recombinant DNA Experiment Safety Committee, Kyoto University for the preparation and use of genetically modified mice for our experiments. The Institute of Laboratory Animals, Graduate School of Medicine, Kyoto University approved our protocol for the experimental manipulation of mice (MedKyo14148). To alleviate the suffering of animals, anesthesia (isoflurane inhalation, induction: 4–5%, maintenance: 2–3%) was used for blood sampling. For euthanasia, carbon dioxide was used.

In the present study, mice were used at different ages for different experiments. Lymphadenopathy in MRL/lpr mice is seen from 8 weeks of age and at 16 weeks of age, the weight of the lymph nodes is almost 100 times that of normal mice [17]. Additionally, symptoms of systemic lupus erythematosus, such as production of anti-DNA antibodies, rheumatoid factor, and gamma globulin, are observed from 12 weeks of age [18]. Moreover, glomerulonephritis, arthritis, and interstitial pneumonia with infiltration of lymphocytes, plasma cells, and histiocytes around the pulmonary vessels [19] have also been reported to occur and develop from the same age. Therefore, 8-week-old were used for analyzing hIgG4 expression, and 12-week-old mice were used for splenocytes phenotyping. We used older mice for tissue phenotyping as the number of cells has been reported to increase with age [19]; however, no difference was observed at 16 weeks.

### Design and construction of h*IGHG4* knock-in mice

To preserve the physiological functions, only the Fc portion of mouse *IgG1* gene was recombined in to the human *IgG4* gene; exons 1, 6, and 7 and introns 1, 5, and 6 of the mIghg1 gene were retained, and exons 2–5 and introns 2–4 were replaced with the hIGHG4 gene to form a chimeric gene (S2 Fig in S1 File). The sequence was inserted into the pBluescript II SK vector. The vector was electroporated into embryonic stem cells (ES) cells of C57BL/6NCrSlc mice, and Southern blotting was performed to select ES clones showing homologous recombination. The recombined ES clones were injected into Balb/c blastocysts, which were transplanted into the uteri of foster mothers. The obtained chimeric mice were then crossed with wild-type C57BL/6NCrSlc mice to produce first-generation (F1) heterozygous mice. F1 mice were in-turn crossed with Cre-transgenic mice to eliminate the neomycin resistance gene. C57/BL6-hIgG4KI mice were thus established and were subsequently backcrossed with MRL/lpr mice for six generations to obtain the MRL/lpr-hIgG4KI mice.

Whole genome sequencing was performed to confirm whether the genes of the MRL/lpr-hIgG4KI mice were replaced by those of MRL/lpr mice. Briefly, DNA was extracted from the tails of MRL/lpr-hIgG4KI and MRL/lpr mice, and whole genome sequencing was performed (Macrogen, Tokyo, Japan). The obtained results were compared with the genome of C57BL/6 mice (GRCm38/mm10, from National Center for Biotechnology Information), and variant call was performed. Single nucleotide polymorphisms (SNPs) on autosomal chromosomes were extracted, and multiple alleles were excluded. From 138,973 SNPs, 100 independent SNPs that were separated from each other by more than 1 Mb were randomly extracted, and the concordance rate of genotypes between MRL/lpr-hIgG4KI and the MRL/lpr-hIgG4KI mice was calculated. Random SNP extraction and concordance rate calculation were performed for 1,000 cycles, and the median concordance rate across 1,000 trials was evaluated.

### Genotyping and quantitative reverse-transcription polymerase chain reaction (PCR)

We extracted DNA from the tails of the MRL/lpr-hIgG4KI mice by ethanol precipitation and determined the mice genotypes by PCR. We used the following primers for PCR: h*IGHG4* (679 bp): forward; 5′-TGAGTAACTCCCAATCTTCTCTCTG-3′, reverse; 5′-CTGACCTGGTTCTTGGTCATCTC-3′. m*Ighg1* (446 bp): forward; 5′-TTCTTCATCCTTAG-TCCCAGAAGTA-3′, reverse; 5′-GTAAAGAGATTGGTTAGCACAGAGG-3′.

To quantify m*Ighg1* and h*IGHG4* gene expression, we extracted mRNA from mice spleens using the RNeasy® Plus Universal Mini Kit (QIAGEN, Hilden, Germany). Subsequently, we synthesized cDNA from the isolated mRNA and quantified the genes by SYBR-green qPCR using the primers mentioned above.

We validated whether the extracellular portion (Exon 5) of h*IGHG4* gene was spliced with the intracellular portion (Exon 6) of m*Ighg1* gene (S3 Fig in S1 File). First, two sets of PCR primers, G1X1F-G1X1R and G1X2F-G1X2R (S3 Fig in S1 File), were used to amplify the regions of the spliced sequence that coded the extracellular and intracellular m*Ighg1* constant regions, respectively. Second, two sets of PCR primers, G4X1F-G1X1R and G4X2F-G1X2R, were used to amplify the regions of the spliced sequence that coded the extracellular h*IGHG4* constant region and the intracellular m*Ighg1* constant region, respectively.

### Enzyme-linked immunosorbent assay (ELISA)

Mice sera were stored at –20°C and thawed before use. We used the following capture antibodies: unlabeled goat anti-mouse IgG1 (Southern Biotech, AL, USA; 1071–01), IgG2a (Southern Biotech; 1080–01), IgG2b (Southern Biotech; 1090–01), IgG2c (Southern Biotech; 1079–01), and IgG3 (Southern Biotech; 1100–01) and rabbit anti-human IgG4 H&L (Abcam, Cambridge, UK, ab86251). These antibodies were diluted 1:100 in phosphate-buffered saline, plated, and stored at 4°C overnight. We used isotype control mouse IgG1 (Southern Biotech; 0102–01), IgG2a (Southern Biotech; 0103–01), IgG2b (Southern Biotech; 0104–01), IgG3 (Southern Biotech; 0105–01), and human IgG4 (Sigma-Aldrich, St. Louis, MO, USA; I4639) as standards. We incubated the standards and samples in 1% bovine serum albumin for 2 h. Following this, they were washed and incubated with the detection antibodies, horseradish peroxidase (HRP)-conjugated goat anti-mouse IgG1 (Sothern Biotech; 1070–05), IgG2a (Southern Biotech; 1080–05), IgG2b (Southern Biotech; 1090–05), and IgG3 (Southern Biotech; 1100–05) at 1:5000 dilution and HRP-conjugated monoclonal HP6023 mouse anti-human IgG4 (Abcam; ab99817) at 1:2000 dilution. Subsequently, a chromogenic reagent was added to the reaction. A turbidimetric immunoassay for the quantification of human IgG4 concentration was outsourced to Japan SRL, Inc (Kumiyama, Japan).

### Immunization of mice

Recombinant human muscle specific tyrosin kinase (MuSK) and ovalbumin (OVA) (final concentration of 1 mg/mL) were emulsified with Complete Freund’s Adjuvant (day 0) and Incomplete Freund’s Adjuvant (day 14) and injected subcutaneously into C57BL/6NCrSlc mice. The sera were sampled on days 0 and 56. Subclass-specific ELISA was performed by 1) plating MuSK and OVA, 2) incubating with mice sera, and 3) detecting with HRP-conjugated anti-mouse IgG1 and anti-human IgG4.

### Flow cytometry

Splenocytes were isolated from the MRL/lpr-hIgG4KI mice and analyzed using BD LSR Fortessa. The following monoclonal antibodies used for flow cytometry as listed in Table 1. Flow cytometry data were analyzed using FlowJo (Tree Star, Ashland, OR, USA). APC-conjugated streptavidin was used to detect biotin-conjugated anti-CD44 antibody. Additionally, unconjugated anti-CD16/32 non- antibody was used to block the Fc receptor.

**Table 1:** Monoclonal antibodies used in flow cytometry in this study.

Manufacturer	Cat. No.	Fluorescence	Antibody	Clone
Biolegend (San Diego, CA, USA)	100733	PerCP/Cy5.5	Mouse CD8a	53–6.7
eBioscience (San Diego, CA, USA)	12-0081-83	PE	Mouse CD8a	53–6.7
Biolegend	100559	BV510	Mouse CD4	RM4-5
Biolegend	100516	APC	Mouse CD4	RM4-5
Biolegend	103236	PerCP/Cy5.5	Mouse CD45R/B220	RA3-6B2
BD (Franklin Lakes, NJ, USA)	553172	PE	Mouse TCRβ	H57-597
Biolegend	100203	FITC	Mouse CD3	17A2
BD	561042	APC/Cy7	Mouse CD3e	145-2C11
Biolegend	152403	FITC	Mouse CD19	1D3
Biolegend	102716	AF647	Mouse CD38	90
Biolegend	142523	BV421	Mouse CD138(Syndecan-1)	281–2
Biolegend	104506	FITC	Mouse CD69	H1.2F3
Biolegend	104408	PE	Mouse CD62L	MEL-14
Biolegend	103003	Biotin	Mouse CD44	IM7
Biolegend	405207	Streptavidin	APC	―
BD	550765	PerCP/Cy5.5	Rat IgG2a,κ	R35-95
Biolegend	400907	PE	Rat IgG2a,κ	HTK888
Biolegend	400511	APC	Rat IgG2a,κ	RTK2758
Biolegend	400907	PE	Hamster IgG	HTK888
Biolegend	400906	FITC	Hamster IgG	HTK888
BD	557662	APC/Cy7	Hamster IgG1κ	A19-3
Biolegend	400526	AF647	Rat IgG2a,κ	RTK2758
Biolegend	400549	BV421	Rat IgG2a,κ	RTK2758
BD	553929	FITC	Rat IgG2a,κ	R35-95

### Histopathology

The mouse spleen, pancreas, kidneys, salivary glands, thyroid gland, stomach, prostate gland, and lymph nodes were frozen in optimal cutting temperature compounds for immunofluorescence staining. We used the following primary antibodies: anti-mouse PE-CD4 (BioLegend, San Diego, CA, USA), FITC-IgG1 (BioLegend) at 1:100 dilutions, and human FITC-IgG4 (Abcam; ab99821) antibody at 1:200 dilution. The tissues were also fixed in 4% paraformaldehyde, embedded in paraffin, and hematoxylin and eosin (H&E) staining and labelled streptavidin biotinylated antibody (LSAB) staining were outsourced to the Center for Anatomical, Pathological and Forensic Medical Researches, Graduate School of Medicine, Kyoto University. We used anti-mouse IgG1 (BioLegend) and anti-human IgG4 (Nichirei Bio-sciences Inc., Tokyo, Japan) as primary antibodies for LSAB.

The severity of inflammation in each organ was evaluated by the following scale (Table 2); each organ was assigned 0–2 or 0–3 points, a score of 0 was reserved for normal observations in each organ.

**Table 2:** Severity scaling of hematoxylin and eosin (H&E)-stained tissue sections.

	1	2	3
Salivary glands	localized inflammatory cell infiltration	diffuse inflammatory cell infiltration	inflammatory cell infiltration with parenchymal destruction
Lung	presence of perivascular inflammatory cell infiltrate	fusion of adjacent perivascular inflammatory cell infiltrates	presence of both inflammatory cell infiltrate in the lung parenchyma with the perivascular infiltrate
Thyroid gland	localized inflammatory cell infiltration	diffuse inflammatory cell infiltration	−
Pancreas	inflammatory cell infiltration around the blood vessels and conduits	inflammatory cell infiltration around the islets of Langerhans along with the features observed in 1	−
Stomach	scattered inflammatory cell infiltrate	prominent inflammatory cell infiltrate	autoimmune gastritis, including atrophy of the gastric fundic glands, fibrosis, and decreased eosinophil count
Kidney	perivascular cell infiltration	obstructive arteritis or phlebitis or granulomatous arteritis	nodules consisting of inflammatory cells in addition to 1 and 2
Prostate gland	mild inflammatory cell infiltration	severe inflammatory cell infiltration	−

Pathological scores were defined using an in-house grading method.

## Results

### Establishing the h*IGHG4* knock-in mice

**Fig 1:**
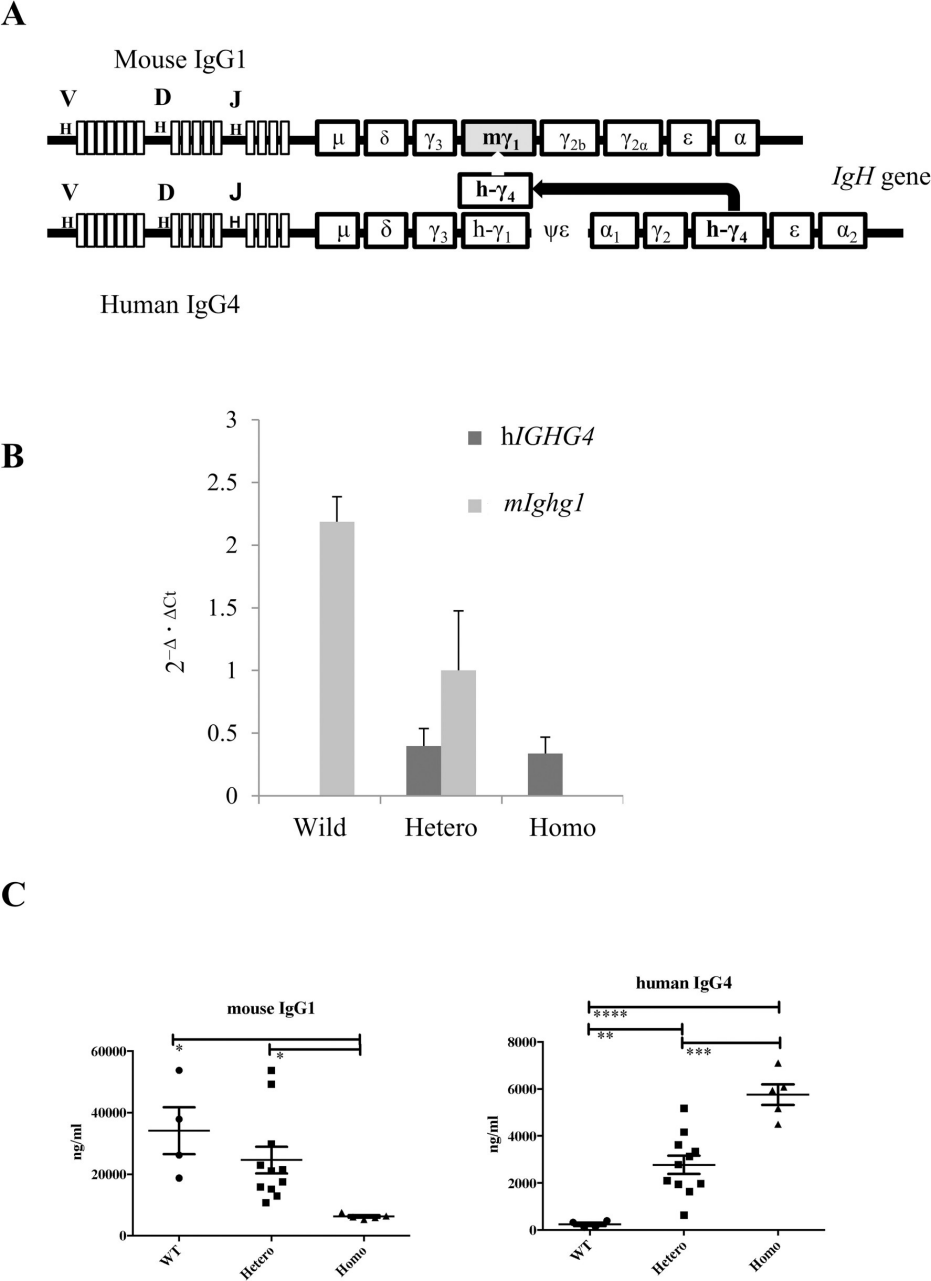
Introduction of the human *IGHG4* gene into mice. A. Genetic design of the human *IGHG4* knock-in mouse. B. Quantitative real-time PCR of h*IGHG4* and m*Ighg1* in splenocytes. Data were calculated by the 2-Δ·ΔCt method using *GAPDH* gene as the control and h*IGHG4* and m*Ighg1* genes as the target genes. C. Serum concentration of mIgG1 and hIgG4 in 5 to 8-week-old mice as assessed by enzyme-linked immunosorbent assay (ELISA). *: P < 0.05, **: P < 0.01, ***: P < 0.001, ****: P < 0.0001 by Bonferroni’s multiple t-test (all pairs). h*IGHG4*, human *IGHG4* gene; m*Ighg1*, mouse *Ighg1* gene. Hetero, heterozygous; Homo, homozygous; WT, wild-type.

We designed a targeting vector carrying a sequence of the h*IGHG4* constant region that replaced the *Ighg1* constant region of the mouse *Igh* gene by homologous recombination (Fig 1A and S1 Fig in S1 File), as was described by Rajewsky et al. [20]. The mouse variable region was retained to preserve VDJ recombination. The h*IGHG4* constant region consists of seven exons and was too long to induce homologous recombination. Thus, we replaced the exons 2–5 and introns 2–4 in h*IGHG4* with their mouse *Ighg1* counterparts. We retained the mouse *Ighg1* exons 6–7 and introns 5–6 to preserve physiological membranous IgG that could be formed by splicing, since exons 6–7 constitute an intracellular constant region. The expressed membrane-type hIgG4 was confirmed to have a chimeric protein that had a mouse IgG1 sequence in the intracellular region (We confirmed it in S3 Fig in S1 File).

Upon mating F1 C57BL/6NCrSlc background mice, we obtained 113 offspring. Subsequently, we determined that the offspring had wild-type, heterozygous, and homozygous genotypes in a ratio of 31:56:26. This ratio was compatible with Mendelian inheritance, suggesting that homozygous mice were not lethal. No abnormalities were observed in the appearance of the mice.

### Measurement of h*IGHG4* mRNA and protein expression in C57BL/6-IgG4KI mice

We quantified the mRNA levels of the m*Ighg1* and h*IGHG4* genes in the spleens of the C57BL/6-IgG4KI mice. We detected the expression of m*Ighg1*, both m*Ighg1* and h*IGHG4*, and h*IGHG4* genes in wild-type, heterozygous, and homozygous mice, respectively (Fig 1B).

To confirm the expression of the chimeric hIgG4 protein in the mice, we amplified the chimeric gene sequence that coded for the extracellular region of the human IgG4 constant region and the intracellular region of the mouse IgG1 constant region by PCR (S3 Fig in S1 File). We prepared cDNA from the mRNA isolated from the spleens of wild-type and homozygous mice. The expression of chimeric membranous hIgG4-mIgG1 was detected using chimeric (hIgG4Ex5-mIgG1Ex6) primers from cDNA derived from homozygous mice. On the contrary, the expression of membranous mIgG1 was detected using allogeneic (mIgG1Ex5-mIgG1Ex6) primers for the cDNA prepared from mRNA extracted from wild-type mice.

Next, to confirm the expression of secretory IgG, we performed ELISA using sera obtained from mice. We detected mIgG1 levels in wild-type mice, both hIgG1 and mIgG4 levels in heterozygous mice, and hIgG4 levels in homozygous mice (Fig 1C), although the concentration of hIgG4 was much lower than that of mIgG1 in heterozygous mice.

However, hIgG4^+^ cells were not detected by pathological analysis and flow cytometry in the spleens of C57BL/6-IgG4KI mice (data not shown).

### Expression of IgG4-type anti-ovalbumin and muscle-specific kinase-specific antibodies was confirmed in C57BL/6-IgG4KI mice

We performed immunization experiments to confirm whether IgG4-type antibodies specific for certain antigens were physiologically produced in the mice. We immunized hIgG4-KI mice with OVA and recombinant human MuSK, as hIgG4-type anti-MuSK antibodies have been reported in myasthenia gravis [7]. In MuSK-immunized wild-type mice, we detected mIgG1-type anti-MuSK antibodies, but not mIgG1-type anti-OVA antibodies. In MuSK-immunized homozygous mice, hIgG4-type anti-MuSK antibodies were detected, but hIgG4-type anti-OVA antibodies were not detected. We obtained contrasting results in OVA-immunized mice, implying that hIgG4-type specific antibodies were produced in the immunized mice (S4 Fig in S1 File).

### MRL/lpr-hIgG4KI mice express secretory and membrane-type IgG4 molecules

**Fig 2:**
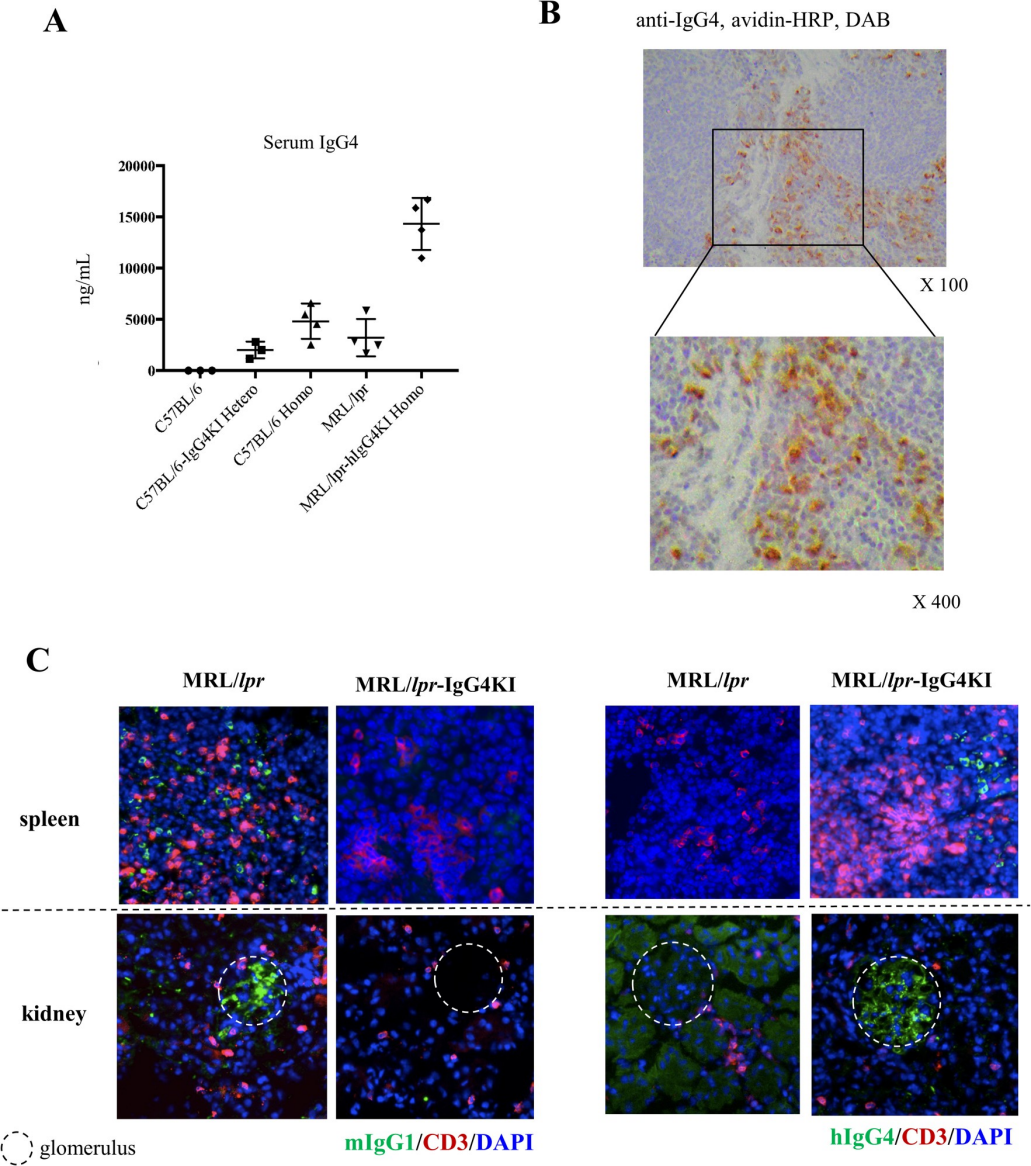
Protein expression of hIgG4 in MRL/lpr-hIgG4KI mice. A. Serum hIgG4 concentration. B. IgG4-positive cells in erythrosplenic medulla of MRL/lpr-hIgG4KI mouse spleen (16-week-old) as assessed by labelled streptavidin biotinylated antibody (LSAB) staining. C. Immunostained IgG4^+^ cells in 8 to 16-week-old mice. Circle indicates glomerulus.

Since the C57BL/6-IgG4KI mice had low hIgG4 protein levels, we generated an MRL/lpr-hIgG4KI mouse model by backcrossing with MRL/lpr mice. The MRL/lpr mouse strain used in the present study (MRL/MpJJmsSlc-lpr/lpr) has an elevated lifespan compared to the MRL/MpJ-Fas^lpr^/J mouse strain (S1 Fig in S1 File). To examine whether the genes of MRL/lpr-hIgG4KI mice were replaced by genes of MRL/lpr, whole genome sequencing of the mice was performed. The median concordance rate of SNPs between MRL/lpr and MRL/lpr-hIgG4KI mice was 95% (S5 Fig in S1 File), using the C57BL/6 mouse genome (GRCm38/mm10) as a reference. Since the MRL/lpr mice have an autoimmune background and hypergammaglobulinemia, we were able to detect high concentrations of hIgG4 in the peripheral blood (Fig 2A and S1 Table in S1 File). We compared the protein expression levels of each IgG subclass in the MRL/lpr mice and MRL/lpr-IgG4KI homozygous mice (S6 Fig in S1 File). While hIgG4 was expressed only in the MRL/lpr-IgG4KI homozygous mice, mIgG1 was only expressed in the MRL/lpr mice. The expression levels of IgG2b and IgG3 did not differ between the two strains and the expression levels of IgG2a in MRL/lpr mice were higher than that of IgG2c in MRL/lpr-IgG4KI mice; IgG2a and IgG2c are alleles. IgG4KI mice were generated in C57BL/6NCrSlc background that has IgG2c, and then backcrossed into MRL/lpr background that has IgG2a. MRL/lpr-IgG4KI mice retain IgG2c, since the knocked-in IgG4 and IgG2c genes are located adjacent to each other.

Subsequently, we pathologically examined the organs resected from the MRL/lpr-hIgG4KI mice and found a number of hIgG4^+^ cells scattered in the erythrosplenic medulla (Fig 2B). Notably, the hIgG4^+^ cells were morphologically plasma cells. Furthermore, immunofluorescence analysis detected hIgG4^+^ cells in the spleens of the homozygous MRL/lpr-hIgG4KI mice, whereas mIgG1^+^ cells were detected in the spleens of the MRL/lpr mice (Fig 2C). We also detected mIgG1 and hIgG4 deposition in the glomeruli of MRL/lpr and MRL/lpr-hIgG4KI mice, respectively; however, we did not detect any mIgG1^+^ or hIgG4^+^ cells in the kidneys (Fig 2C).

### MRL/lpr-hIgG4KI mice exhibit enhanced inflammation

**Fig 3:**
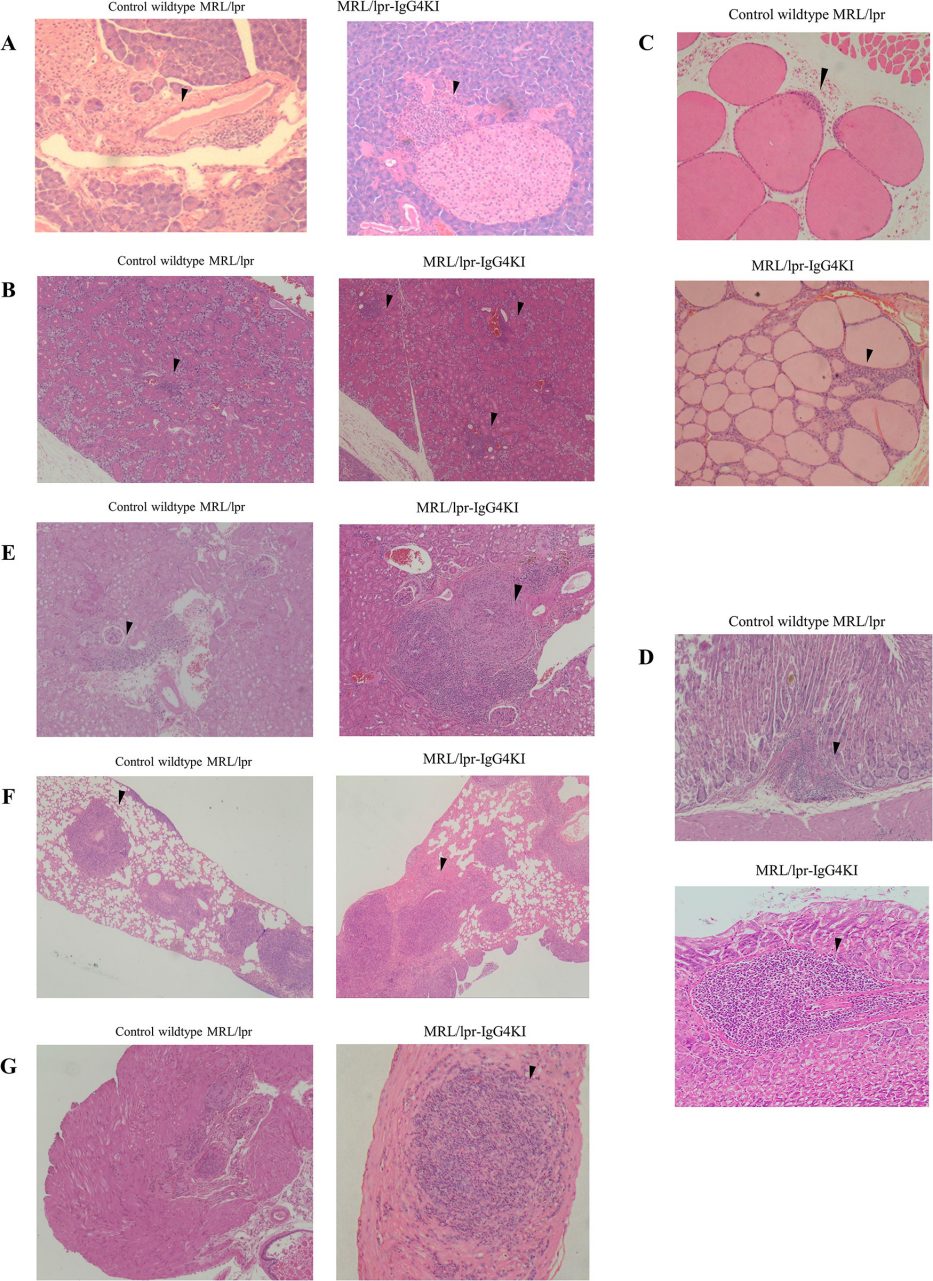
Hematoxylin and eosin (H&E)-stained tissue sections in MRL/lpr-hIgG4KI homozygous mice. A. Marked inflammatory cell infiltration around blood vessels and, partially, inflammatory cell infiltration around islets of the pancreas. B. Diffuse inflammatory cell infiltration in the salivary gland. C. Diffuse inflammatory cell infiltration around follicles of the thyroid gland. D. Prominent inflammatory cell infiltration in submucosa of the stomach. E. Marked perivascular inflammatory cell infiltration in the kidney. F. Marked perivascular inflammatory cell infiltration in the lung. G. Marked inflammatory cell infiltration in the prostate gland.

As we did not observe any abnormal features in any organ of the C57BL/6-IgG4KI mice, we compared the organ specimens of the MRL/lpr-hIgG4KI and MRL/lpr mice by H&E staining. At 16 weeks, the MRL/lpr-hIgG4KI and MRL/lpr mice did not have any apparent differences in their organs. However, at 52 weeks, organs of the MRL/lpr-hIgG4KI mice displayed more severe inflammation than those of the MRL/lpr mice (Fig 3 and Table 3). Additionally, the serum IgG-type anti-DNA antibody titer was lower in the MRL/lpr-hIgG4KI mice than in the MRL/lpr mice (S6 Fig in S1 File).

**Table 3:** Scoring of hematoxylin and eosin (H&E)-stained tissue sections.

	MRL/lpr (n = 3)	MRL/lpr IgG4KI (n = 3)	p*
Salivary glands	1.33 ± 0.33	2.33 ± 0.33	0.10
Thyroid gland	1	2	-
Lung	2.33	2.33	-
Kidney	2 ±0.58	2.67 ± 0.33	0.37
Pancreas	1	2	-
Stomach	1± 0.58	2.67± 0.33	0.08
Prostate gland	1 ± 0	1.67 ± 0.33	0.12
Total score	8.67 ± 1.76	15.67 ±1.20	0.04

See Materials and Methods for the evaluation of the severity. Data are presented as the mean ± standard deviation. N = 3 in each group. p values were calculated by Welch’s test. Tests were not performed because the scores for the pancreas and the thyroid gland were the same within the group, and the average for the lung were the same between the groups.

### Analysis of splenocytes in MRL/lpr-hIgG4KI mice

**Fig 4:**
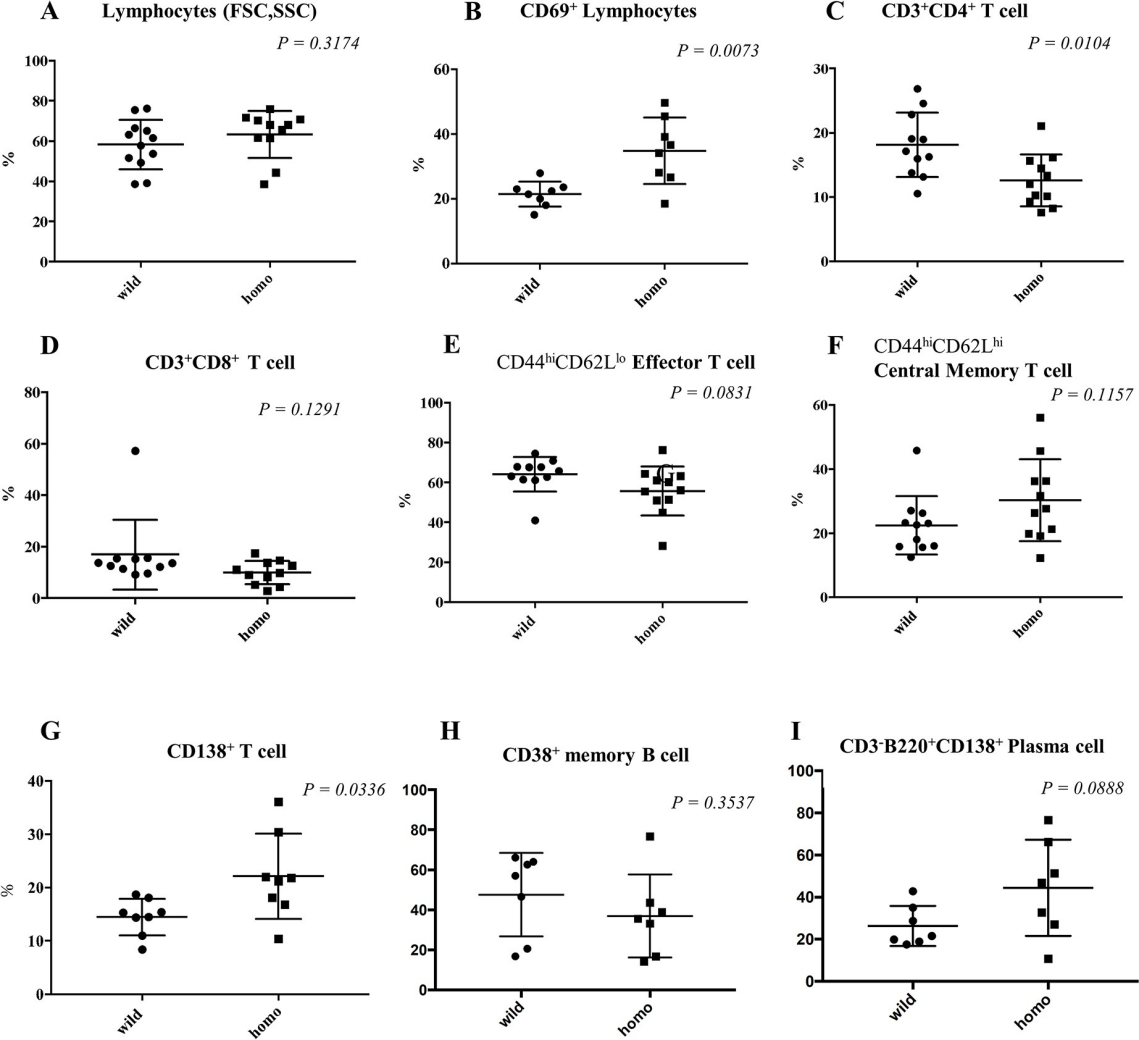
Analysis of splenocytes in MRL/lpr-IgG4KI mice. A. Percentages of lymphocytes in splenocytes; FSC, Forward Scatter; SSC, Side Scatter. B. Percentage of activated CD69^+^ cells in lymphocytes. C. Percentage of CD4^+^ SP T cells in CD3^+^B220^−^ cells. D. Percentage of CD8^+^ SP T cells in CD3^+^B220^−^ cells. E. Percentage of CD4SP effector (CD44^+^CD62L^−^) T cells in CD3^+^CD4^+^B220^−^ cells. F. Percentage of CD4SP central memory (CD44^+^CD62L^+^) T cells in CD3^+^CD4^+^B220^−^ cells. G. Percentage of CD138^+^ cells in lymphocytes. H. Percentage of CD38^+^ memory B cells in CD3^−^B220^+^ cells. I. Percentage of CD138^+^ plasma cells in CD3^−^B220^+^ cells in the spleens of 18 to 25-week-old MRL/lpr (wild-type) and MRL/lpr-hIgG4KI homozygous mice. P < 0.05 was considered statistically significant (Welch’s test). Wild-type MRL/lpr: male: n = 2, female: n = 6; homozygous MRL/lpr-IgG4KI, male: n = 3, female: n = 5. The gating strategies are shown in S7 Fig in S1 File.

Since the MRL/lpr mice are generally characterized by infiltration of inflammatory cells in tissues, we examined which splenocytes were activated in MRL/lpr-hIgG4KI homozygous mice. Absolute splenocyte counts were significantly higher in the MRL/lpr-IgG4KI mice (4.18 ± 0.54 × 10^8^/body) than in the wild-type MRL/lpr mice (2.03 ± 0.19× 10^8^/body) (*P* = 0.0041). No difference was observed in lymphocyte percentages between the two strains of mice (Fig 4A and Gating strategies were shown in S7 Fig in S1 File). We found that the percentage of CD3^+^B220^−^CD4^+^ single-positive (SP) cells was significantly decreased in the MRL/lpr-IgG4KI mice (Fig 4C). In the CD4SP population of the MRL/lpr-hIgG4KI mice, there tended to be an increased population of central memory (CM) T cells (Fig 4F) and decreased population of effector T cells (Fig 4E) than in the CD4SP population in MRL/lpr mice. The percentage of CD8SP cells (Fig 4D), the ratios of naïve, CM, and effector cells did not differ between the two mice strains (S8 Fig in S1 File). The percentage of C3^+^CD4^−^CD8^−^B220^+^ cells, which are a characteristic of MRL/lpr mice, did not differ between the two strains (S8 Fig in S1 File).

The percentage of CD3^+^B220^+^CD138^+^ T cells, which is reported to be associated with autoantibody production in the MRL/lpr mice [21], was significantly increased in MRL/lpr-hIgG4KI mice (Fig 4G). We also found that the percentage of CD69^+^ lymphocytes (selected by forward and side scatter) was significantly increased in MRL/lpr-hIgG4KI mice (Fig 4B). The percentage of CD69^+^CD4SP and CD69^+^CD8SP T cells did not differ between the MRL/lpr-hIgG4KI and MRL/lpr mice. On the other hand, the percentage of B220^+^CD69^+^ cells was significantly higher in MRL/lpr-hIgG4KI mice than that in the MRL/lpr mice (S9 Fig in S1 File).

Analysis of B cell lineage (CD3^−^B220^+^) revealed a decrease in the percentage of CD3^−^B220^+^CD38^+^ memory B cells (Fig 4H) in MRL/lpr-hIgG4KI mice, whereas there was an increase in the percentage of CD3^−^B220^+^CD138^+^ plasma cells in MRL/lpr-hIgG4KI mice (Fig 4I).

## Discussion

The level of IgG4, a minor IgG subclass with a unique structure, is elevated in allergic diseases [5]. IgG4-type autoantibodies are pathogenic in several diseases [6–9]. However, the functions of IgG4 are still not clear. In this study, we generated knock-in mice, wherein the h*IGHG4* constant region was replaced with the m*Ighg1* constant region, to investigate the role of hIgG4.

Here, we observed high hIgG4 expression in the sera, hIgG4 deposition in the kidney, and hIgG4-positive plasma cells in the spleen of MRL/lpr-hIgG4KI mice. h*IGHG4* knock-in mice also exhibited exacerbated inflammation when crossed with MRL/lpr mice. In addition, the percentages of CD3^+^B220^+^CD138^+^ T cells and CD3^+^B220^+^CD69^+^ T cells were significantly increased in MRL/lpr-hIgG4KI mice. Although hIgG4 is considered to have protective characteristics in some conditions [22], we discovered that it mediated increased higher exacerbation of inflammation in MRL/lpr-hIgG4KI mice than in MRL/lpr mice. We hypothesize four possible reasons for this observation.

First, hIgG4 has been demonstrated to be not only a protective, but also a pathogenic antibody. Shiokawa et al. reported that administering serum IgG obtained from patients with IgG4-RD induced pancreatitis in new-born BALB/c mice [15]. The pathogenic component of the IgG was later proven to be an anti-laminin-E511 antibody [23]. Shiokawa et al. [15] observed that the transfer of hIgG4 fraction was pathogenic; although the degree of inflammation was milder than that induced by transfer of hIgG1 fraction. Sasaki et al. demonstrated [24] that OVA-specific hIgG4, as well as other subclasses, can induce pancreatitis when OVA-specific cytotoxic T lymphocytes are simultaneously transferred into RIP-mOVA mice, wherein the *OVA* gene was expressed specifically in the pancreatic islets. In addition, a previous study showed that serum fucosylated IgG4 level was significantly elevated in patients with IgG4-RD compared with that in healthy controls. Furthermore, it was implied that the pathogenicity of the fucosylated IgG4 was mediated by the activation of complement cascade [25]. These reports suggest that hIgG4 can be autoreactive, has some complement-activating functions, and may have been involved in the pathogenesis observed in our transgenic mice.

Furthermore, we speculate that mIgG1 deficiency in the h*IGHG4* KI homozygous mice may have worsened inflammation. Strait et al. analyzed the role of mIgG1 both in mice immunized with goat-anti-mouse IgD (GaMD; a complement- and FcγR-independent model) and mice with collagen-induced arthritis (CIA; a complement- and FcγR-dependent model). They reported that mIgG1 deficiency aggravated the severity of cryoglobulinemic glomerulonephropathy in GaMD-immunized mice [22]. This is because mIgG1 acts as a neutralizing antibody that inhibits the formation of cryoglobulin, thereby promoting the clearance of immune complexes that are formed in the kidney. Next, CIA was exacerbated in the C57BL/6-background IgG1-deficient mice, whereas IgG1 deficiency did not exacerbate CIA in the BALB/c background mice [26]. Strait et al. [26] demonstrated that IgG2a/c (2a; BALB/c, 2c; C57BL/6NCrSlc) was correlated with disease severity and responsible for the pathogenesis of CIA through the activation of the complement system and FcγR. Since BALB/c mice had high expression of IL-4, low expression of IFN-γ, and low production of IgG2a (one-fourth that of IgG2c in C57BL/6NCrSlc mice), IgG1 deficiency did not affect the severity of the arthritis in the BALB/c mice. In our model, we assumed that the lack of mIgG1 did not affect the pathophysiology of the MRL/lpr-hIgG4KI mice, because we observed no increase in serum IgG2c levels in MRL/lpr-hIgG4KI mice compared with the IgG2a levels in MRL/lpr mice. This result is similar to the observation of BALB/c CIA mice by Strait et al. [26]. Therefore, it is likely that increased levels of hIgG4 are responsible for enhanced inflammation in MRL/lpr-hIgG4KI mice.

Additionally, we discuss the possibility that the exacerbated inflammation occurred because of decreased FcγRIIb expression. The MRL/lpr-hIgG4KI homozygous mice did not express mIgG1, which is the main Ig subclass that binds to FcγRIIb. FcγRIIb deficiency in MRL/MpJ mice causes glomerulonephritis by promoting germinal centre formation, plasma cell infiltration, and anti-DNA antibody production [27]. However, in our analysis, the germinal centers in the spleens of MRL/lpr-hIgG4KI mice were destroyed compared with those of MRL/lpr-wild-type mice (S10 Fig in S1 File). Therefore, the pathophysiology might be different in our MRL/lpr-hIgG4KI mice compared with FcγRIIb-knockout MRL/MpJ mice whose germinal centre numbers and size were increased [27]. Additionally, FcγRIIb levels may be decreased in MRL/lpr mice, as their expression is downregulated by IFNγ and TNF-α [28], the levels of which are elevated in MRL/lpr mice, and these are prominent features of an autoimmune syndrome in the MRL/lpr mice [29, 30]. However, this issue is not limited to the affinity between the IgG subclasses and Fcγ receptors. In patients with cancer, although the affinity of the IgG1-type and IgG4-type anti-chondroitin sulphate proteoglycan 4 antibodies to FcγRI is similar, down-stream signal transduction after their binding differs between the two antibody types [31]. Therefore, we consider that the decreased FcγRIIb signal in the absence of mIgG1 might not enhance inflammation in MRL/lpr-hIgG4KI mice.

Finally, the CD3^+^B220^+^CD138^+^ T cells possibly play a role in aggravating inflammation in MRL/lpr-hIgG4KI mice. MRL/lpr mice have CD3^+^B220^+^CD4^-^CD8^-^ double-negative T cells, a characteristic feature of this strain. Among them, CD3^+^B220^+^CD138^+^ T cells are the CM type that remain in the lymph nodes and promote the differentiation of autoantibody-producing B cells. Akkoyunlu et al. demonstrated that these T cells in the MRL/lpr mice stimulate autoantibody-producing B cells *in vitro* [21]. In the present study, the percentage of CD3^+^B220^+^CD138^+^ T cells was higher in MRL/lpr-hIgG4KI mice than that in MRL/lpr mice. Furthermore, inflammation was more severe in MRL/lpr-hIgG4KI mice than that in MRL/lpr mice. The percentage of B220^+^CD69^+^ activated lymphocytes, which we confirmed were CD3^+^B220^+^CD138^+^ cells, was also higher in MRL/lpr-hIgG4KI mice than in MRL/lpr mice. To summarize, hIgG4 and CD3^+^B220^+^CD138^+^ T cells may be associated with aggravated inflammation observed in the transgenic mice.

Nevertheless, the present study has certain limitations. To understand the pathophysiology of the MRL/lpr-hIgG4KI mice, we need to determine the following: 1) the binding strength of the genetically manipulated hIgG4 with complement proteins and FcγRs, 2) whether the binding of hIgG4 to FcγRs can induce intracellular signaling, and 3) the types of cells that express FcγRs and receive hIgG4 signals. Another limitation is the small sample size as it is too small to make conclusion. Therefore, further study is needed to validate our results.

Our established mouse model can be used to elucidate the pathophysiology of allergic diseases, IgG4-RD, and IgG4-type antibody-related diseases, including pemphigus [6], MuSK myasthenia gravis [7, 15], idiopathic membranous nephropathy [9], and thrombotic thrombocytopenia [8]. IgG4-type autoantibodies are pathogenic in some autoimmune diseases, whereas IgG4 is associated with protection against food allergies [10, 11] by promoting the function of immunoregulatory M2 macrophages [32]. We have demonstrated that IgG4-type anti-OVA and IgG4-type anti-MuSK antibodies can be induced in our transgenic mice. Although we backcrossed them with MRL/lpr mice in the present study, it is also possible to select other mice strains to establish various autoimmune disease models.

### Conclusions

In conclusion, our study demonstrated that MRL/lpr-hIgG4KI mice had high hIgG4 concentrations in sera, appearance of IgG4^+^ plasma cells, and increased number of CD3^+^B220^+^CD138^+^ T cells in the spleen. The KI mice also had aggravated inflammation in organs, such as the salivary glands and stomach. The MRL/lpr-hIgG4KI mouse model may be useful to study IgG4-RD, IgG4-type antibody-related diseases, and allergic diseases.

## Supporting information

S1 File (PDF)

S1 Raw images Original gel photographs of S3 Fig in S1 File. (PDF)
