# Supplementary material for: Increased number of T cells and exacerbated inflammatory pathophysiology in a human IgG4 knock-in MRL/lpr mouse model
Source: PLoS One. 2023 Feb 10;18(2):e0279389. doi: 10.1371/journal.pone.0279389 (PMC9916631; doi:10.1371/journal.pone.0279389)
Supplement: S1 File — (PDF) [file pone.0279389.s001.pdf]

# Supplementary Materials

## Title:

Increased number of T cells and exacerbated inflammatory pathophysiology in a human IgG4 knock-in MRL/lpr mouse model

**Authors:**

Yoshie Gon, Tsugumitsu Kandou, Tatsuaki Tsuruyama, Takeshi Iwasaki, Koji Kitagori, Kosaku Murakami, Ran Nakashima, Shuji Akizuki, Akio Morinobu, Masaki Hikida, Tsuneyo Mimori, and Hajime Yoshifuji

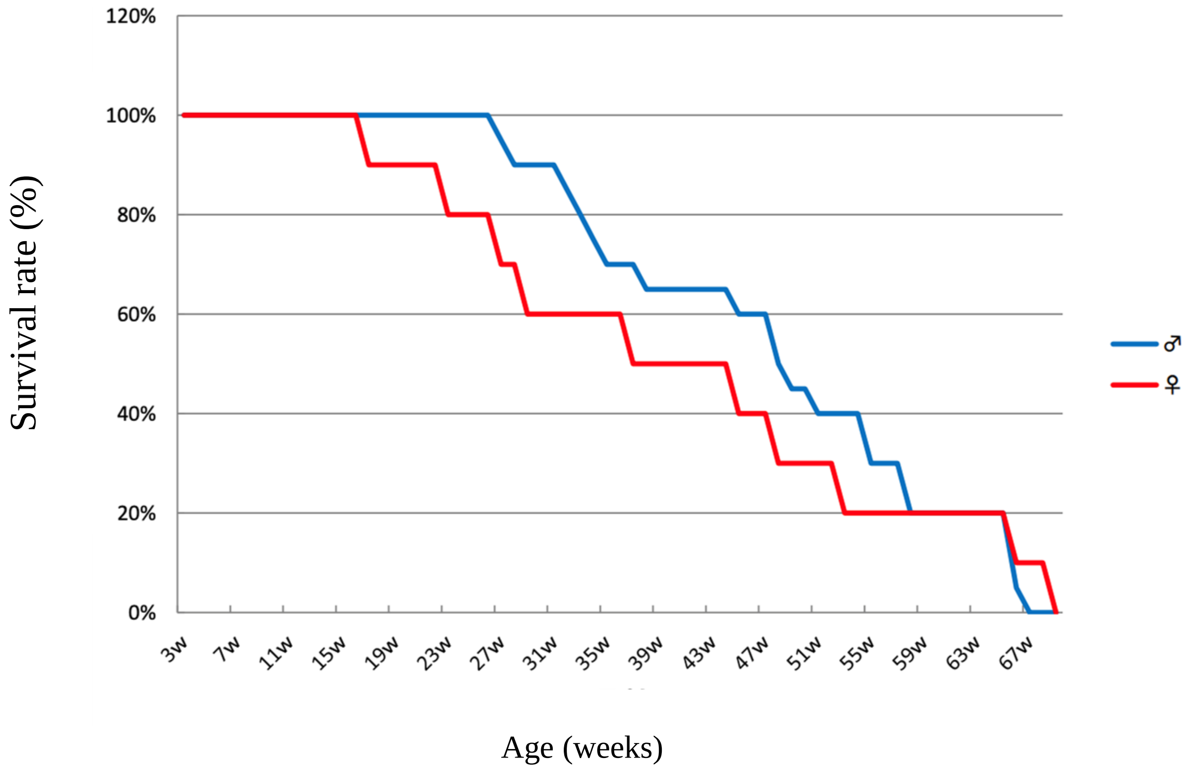


**S1 Fig. Survival curve of MRL/lpr mice used in this study.** The graph was obtained from Japan SLC, Inc. (Shizuoka, Japan)

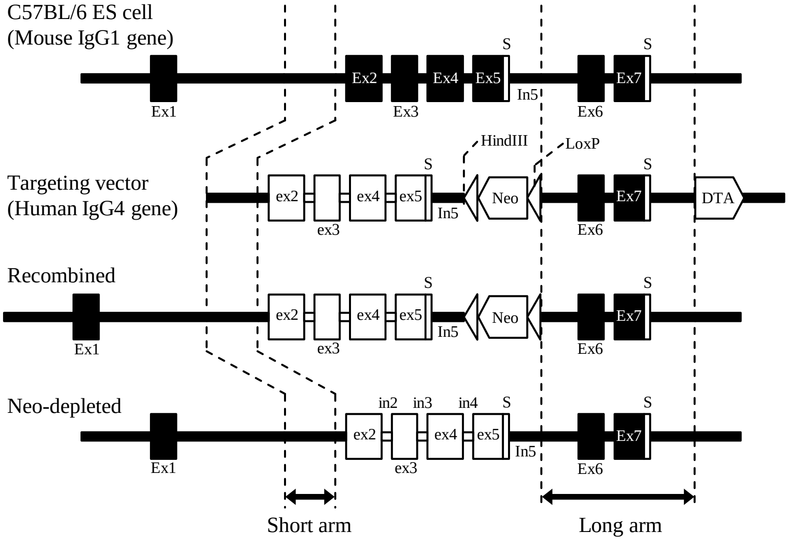


**S2 Fig. Design of the targeting vector.** Mouse IgG1 heavy chain constant region (Ex2–Ex5) was replaced by human IgG4 counterpart (ex2–ex5). Note that IgH genes have two stop codons (S) to form secretory (Ex1–Ex5) and membranous (Ex1–-Ex7) immunoglobulins by splicing. Mouse IgG1 exon 1 remained untouched since it was far from the other exons. We untouched mouse IgG1 exons 6 and 7 to preserve the mouse original intracellular signaling. We also untouched mouse IgG1 intron 5 (In5) to preserve usual splicing. DTA, diphtheria toxin A; Ex1–Ex7, mouse IgG1 exon 1 to exon 7; ex2–ex5, human IgG4 exon 2 to exon 5; In5, mouse IgG1 intron 5; in2–in4, human IgG4 intron 2 to intron 4; Neo, neomycin resistance gene; S, Stop codon.

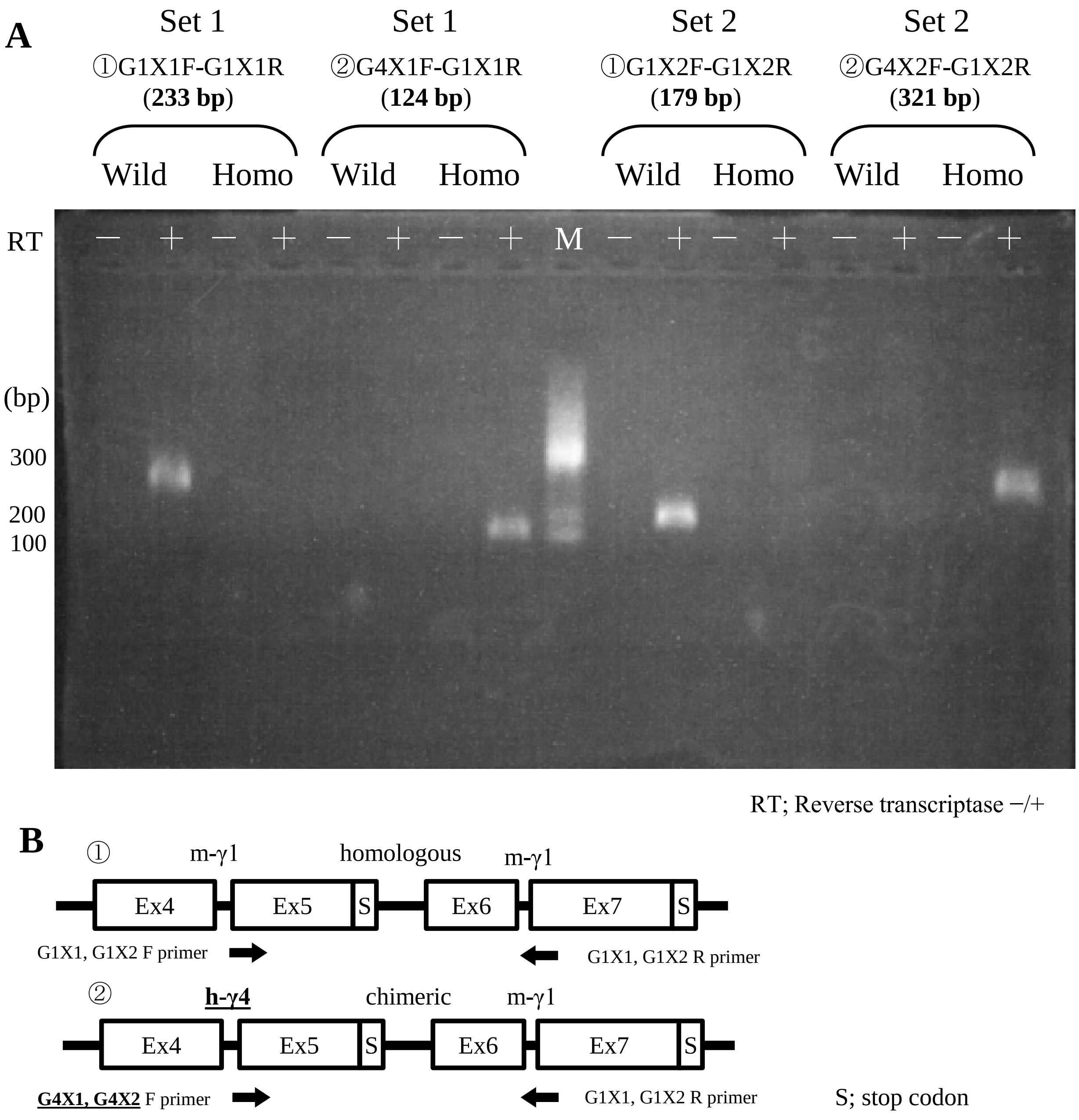


**S3 Fig. Confirmation of chimeric splicing from human IgG4 sequences (Exon 2–5) to mouse IgG1 sequences (Exon 6–7) by PCR. A.** Amplified PCR products derived from tails of wild-type (C57BL/6NCrSlc) and IgG4KI homozygous mice. **B.** Design of the primers:
Set 1: ① [G1X1F] CCAAAGGCAGACCGAAGGCT, ② [G4X1F] GGTGGCAGGAGGGGAATGTC, [G1X1R] TGGGCCTCAGCACAGGTCTC.
Set 2: ① [G1X2F] ACTGGGAGGCAGGAAATACT, ② [G4X2F] GTCAAAGGCTTCTACCCCAG, [G1X2R] GAAGAGGCTGATGAAGATGG.

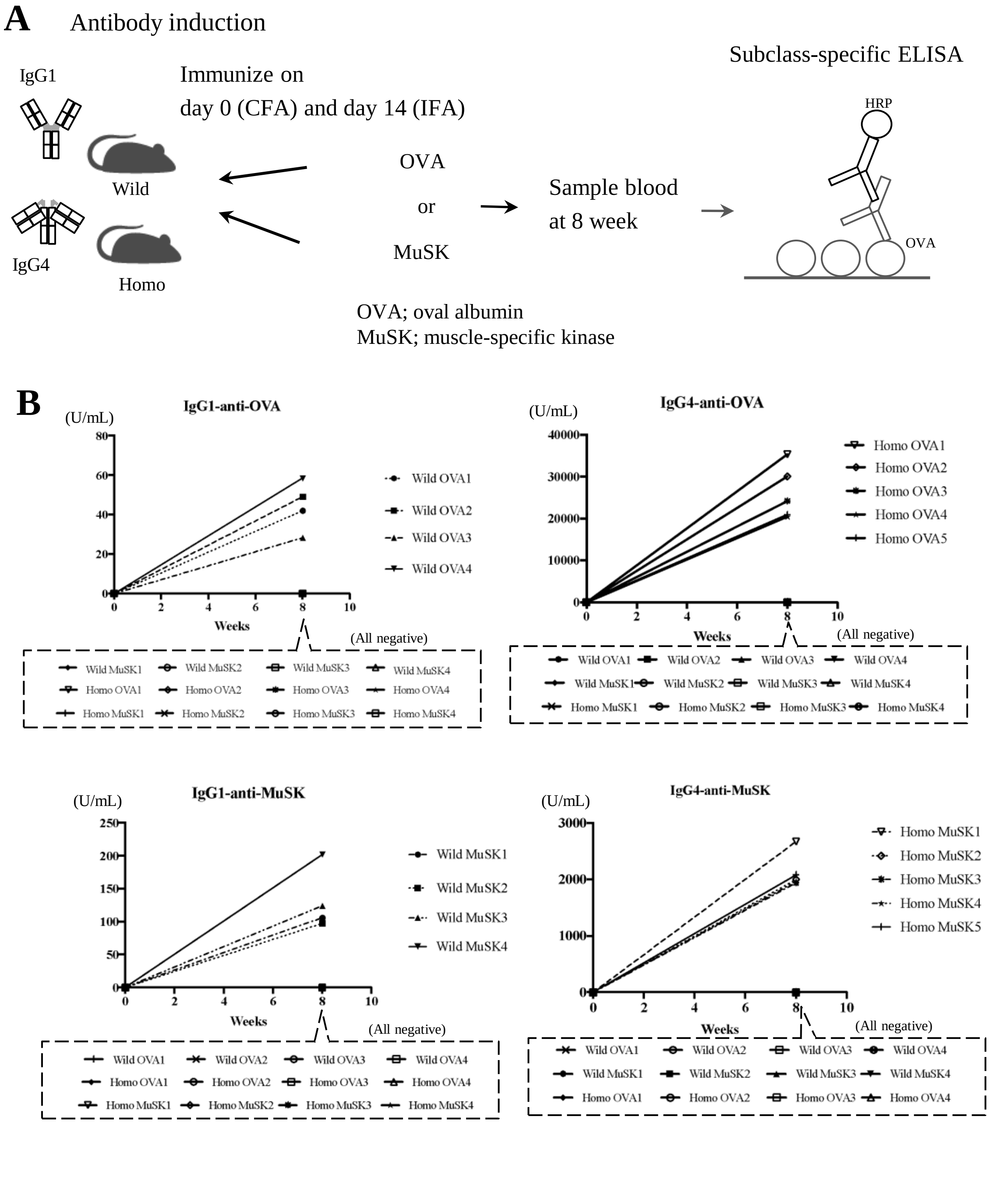


**S4 Fig. IgG4-type autoantibodies induced in IgG4KI mice. A.** Immunization of wild-type (C57BL/6NCrSlc) and IgG4KI homozygous mice with OVA or MuSK. **B.** Subclass-specific ELISA for antibodies to OVA and MuSK.

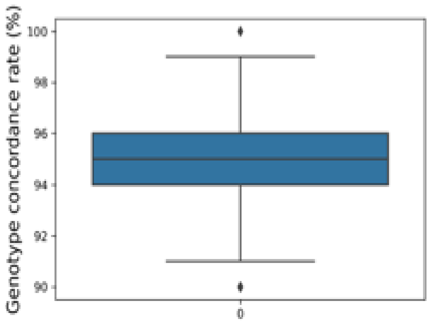


**S5 Fig. Concordance rate of SNPs between MRL/lpr and MRL/lpr-hIgG4KI mice, using C57BL/6 mouse genome as a reference.** Whole genome sequencing was performed using tail DNA of MRL/lpr and MRL/lpr-hIgG4KI mice. The sequencing results were mapped to the genome of C57BL/6 mice (GRCm38/mm10), and variant calling was performed. From 138,973 autosomal SNPs multiple alleles were excluded and 100 independent SNPs that were separated from each other by more than 1 Mb were randomly extracted, and the concordance rate of genotypes between MRL/lpr-hIgG4KI and MRL/lpr-hIgG4KI mice was calculated. Random SNP extraction and concordance rate calculations were performed for 1,000 cycles and the median concordance rate across 1,000 trials was 95%. Data is presented as a box-whisker plot with dots indicating outliers.

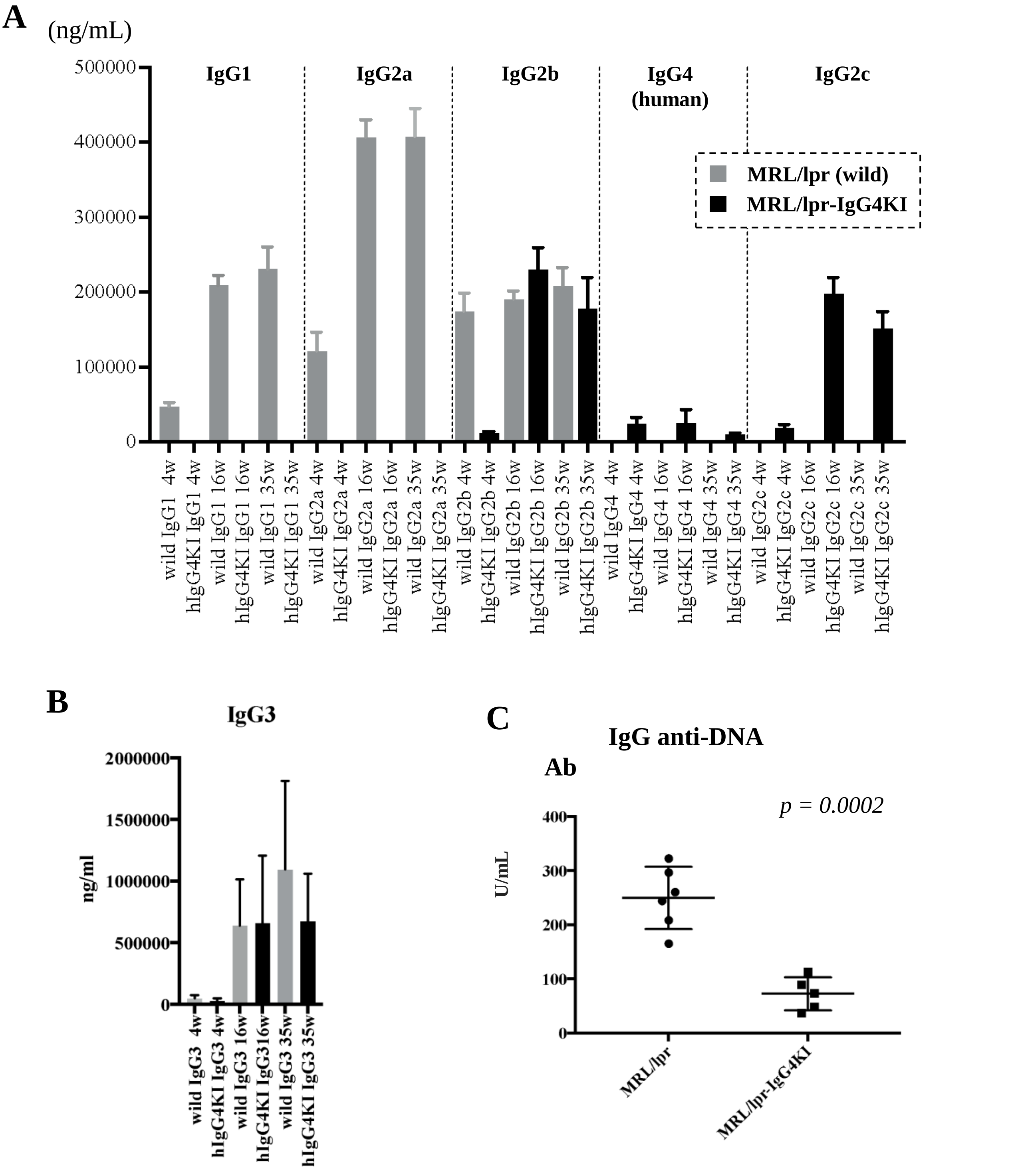


**S6 Fig. Concentration of each IgG subclass (A) and IgG3 (B), and titers of IgG-type anti-DNA antibodies (C) in MRL/lpr (wild-type) and MRL/lpr-IgG4KI homozygous mice measured by ELISA.** Note that IgG2a and IgG2c are alleles. IgG4KI mice were generated in C57BL/6NCrSlc background that has IgG2c, and then backcrossed into MRL/lpr background that has IgG2a. MRL/lpr-IgG4KI mice still retain IgG2c. The titers of anti-DNA Ab were expressed as relative values when the standard sera derived from a NZB/W-F1 mouse was set as 100 U/mL. P value was calculated by Welch’s test.

## A. T cells

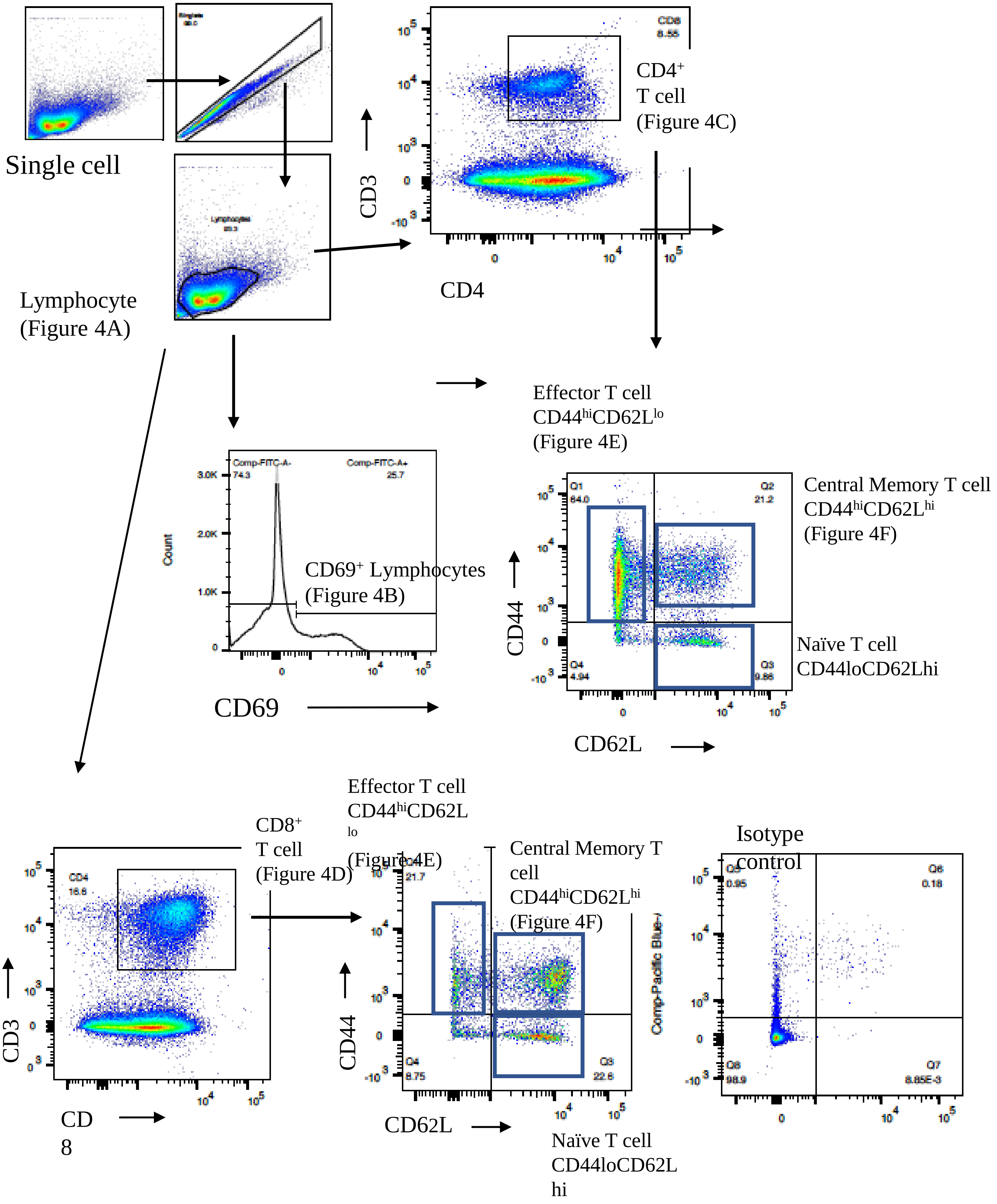


**S7 Fig. Gating strategies in flow cytometry analysis (Fig. 4, S8, and S9).**

**A.** Gating strategy for T cells.

## B. B cells

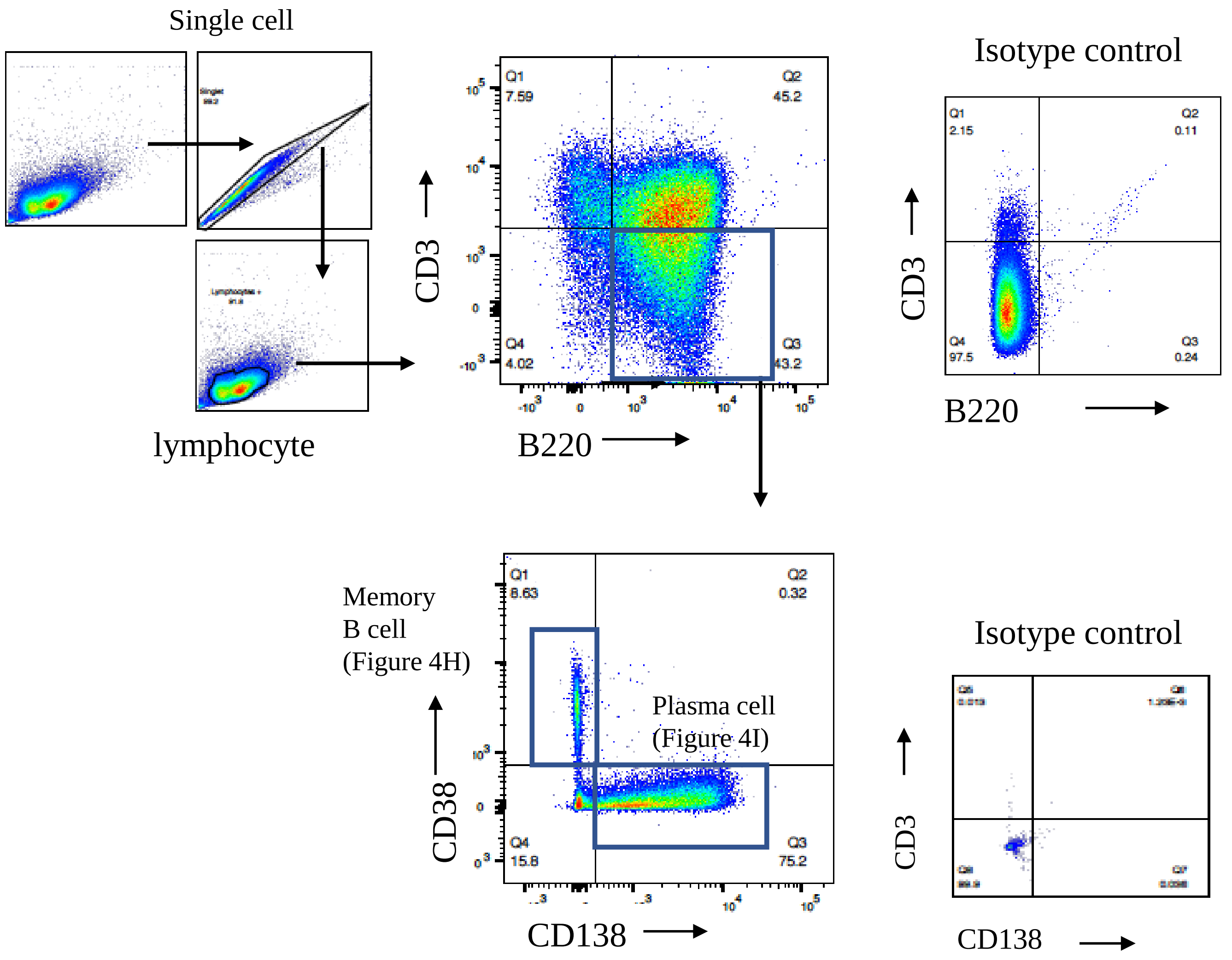


**S7 Fig. Gating strategies in flow cytometry analysis (Fig. 4, S8, and S9).**
**B.** Gating strategy for B cells.

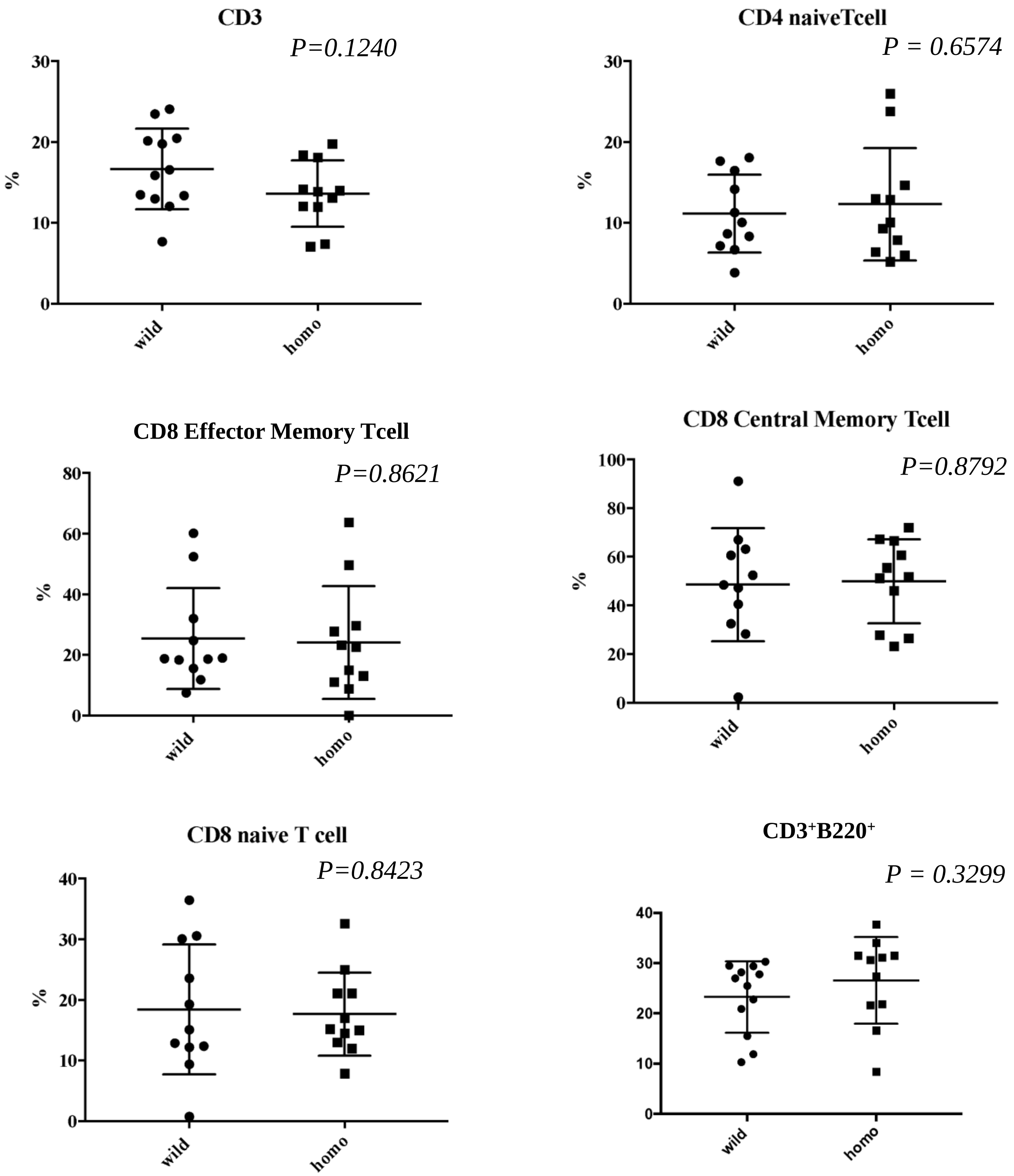


**S8 Fig**. **Analysis of splenocytes in MRL/lpr-IgG4KI mice by FCM.** Percentage of $CD3^{+}B220^{-}$ cells in lymphocytes, CD4SP naïve ($CD44^{-}CD62L^{+}$) T cells in $CD3^{+}CD4^{+}B220^{-}$ cells, CD8SP effector ($CD44^{+}CD62L^{-}$), CD8SP central memory ($CD44^{+}CD62L^{+}$), and CD8SP naïve ($CD44^{-}CD62L^{+}$) T cells in $CD3^{+}CD8^{+}B220^{-}$ cells, and $CD3^{+}B220^{+}$ cells in lymphocytes in spleens of 18 to 25-week-old MRL/lpr (wild-type) and MRL/lpr-IgG4KI homozygous mice. P values were calculated by Welch's test.

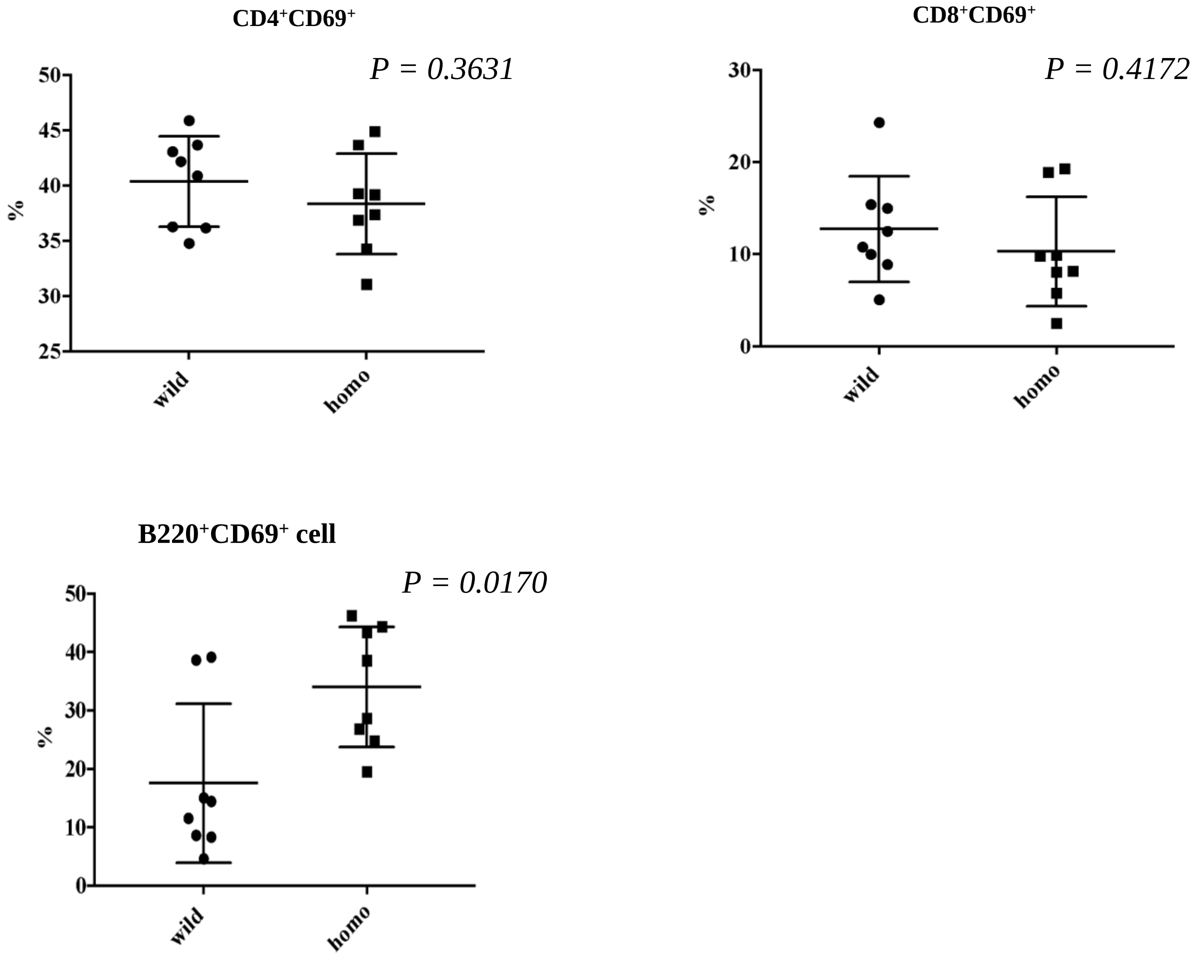


**S9 Fig**. **Analysis of splenocytes in MRL/lpr-IgG4KI mice (continued).** Percentage of activated $CD69^+$ $CD4^+$ SP in $CD3^+CD4^+$ cells, activated $CD69^+$ $CD8^+$SP in $CD3^+CD8^+$ cells and $B220^+CD69^+$ cells in $B220^+$ cells in spleens of 18 to 25-week-old MRL/lpr (wild-type) and MRL/lpr-IgG4KI homozygous mice was measured by FCM. P values were calculated by Welch’s test.

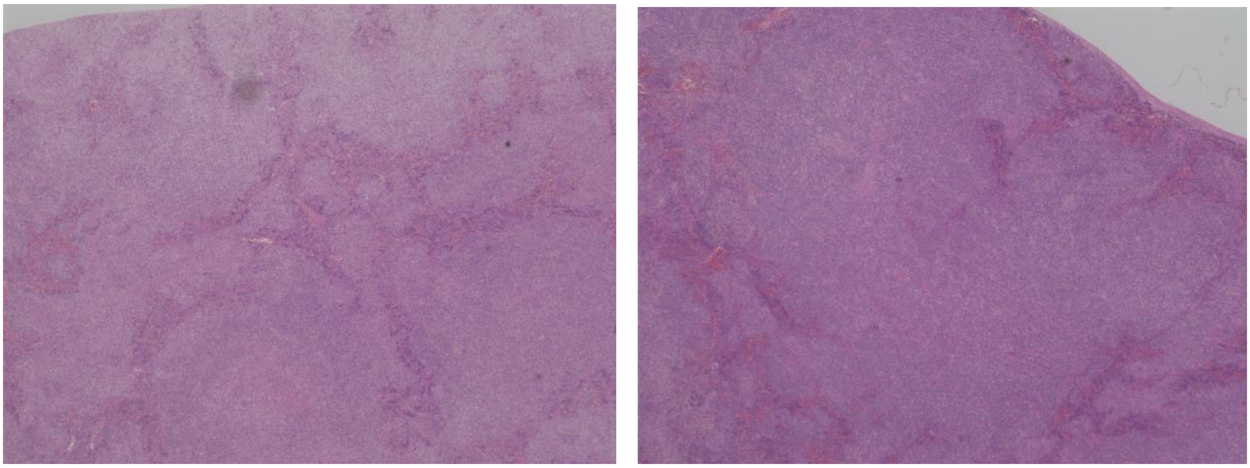

MRL/lpr wild-type mice MRL/lpr-hIgG4KI homozygous mice

**S10 Fig. Hematoxylin and eosin (H&E)-stained tissue sections of spleen in MRL/lpr-hIgG4KI homozygous mice and MRL/lpr wild-type mice.** The follicular structures were discarded and fused in the spleen of MRL/lpr-hIgG4KI mouse compared with those in the spleen of MRL/lpr wild-type mice.

**Supplementary Table S1. Serum hIgG4 concentration in mice**

| | hIgG4 (mg/dL) |
|---|---|
| C57BL/6 | Not detected |
| C57BL/6-hIgG4KI | 7.0 |
| MRL/lpr | Not detected |
| MRL/lpr-hIgG4KI | 289 |

High concentration of hIgG4 was detected in MRL/lpr-hIgG4KI mice by turbidmetric immunoassay.
