# Supplementary figures and images for: Increased number of T cells and exacerbated inflammatory pathophysiology in a human IgG4 knock-in MRL/lpr mouse model
Source: PLoS One. 2023 Feb 10;18(2):e0279389. doi: 10.1371/journal.pone.0279389 (PMC9916631; doi:10.1371/journal.pone.0279389)

2016.02.18 14:00

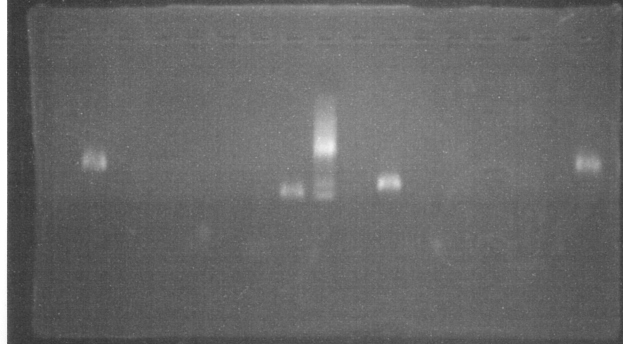

2016.02.18 14:00

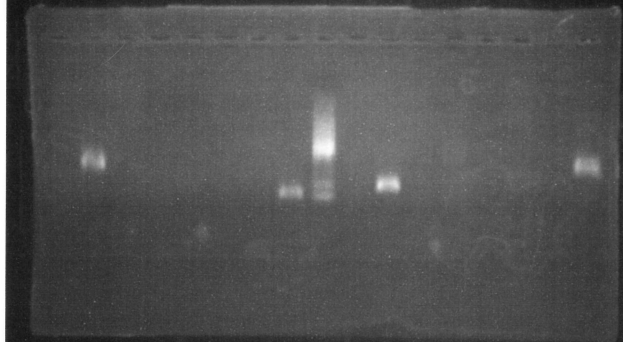

2016.02.18 14:00

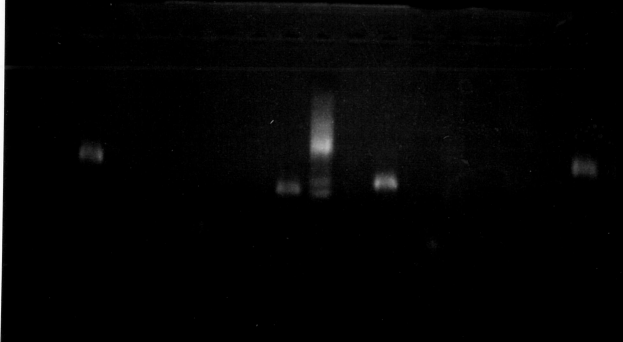

Supplement: S1 Raw images — (PDF) [file pone.0279389.s002.pdf]
